# Multi-Model Domain Adaptation for Diabetic Retinopathy Classification

**DOI:** 10.3389/fphys.2022.918929

**Published:** 2022-07-01

**Authors:** Guanghua Zhang, Bin Sun, Zhaoxia Zhang, Jing Pan, Weihua Yang, Yunfang Liu

**Affiliations:** ^1^ Department of Intelligence and Automation, Taiyuan University, Taiyuan, China; ^2^ Graphics and Imaging Laboratory, University of Girona, Girona, Spain; ^3^ Shanxi Eye Hospital, Taiyuan, China; ^4^ Department of Materials and Chemical Engineering, Taiyuan University, Taiyuan, China; ^5^ Affiliated Eye Hospital, Nanjing Medical University, Nanjing, China; ^6^ The First Affiliated Hospital of Huzhou University, Huzhou, China

**Keywords:** diabetic retinopathy classification, multi-model, domain adaptation, convolutional neural network, deep learning

## Abstract

Diabetic retinopathy (DR) is one of the most threatening complications in diabetic patients, leading to permanent blindness without timely treatment. However, DR screening is not only a time-consuming task that requires experienced ophthalmologists but also easy to produce misdiagnosis. In recent years, deep learning techniques based on convolutional neural networks have attracted increasing research attention in medical image analysis, especially for DR diagnosis. However, dataset labeling is expensive work and it is necessary for existing deep-learning-based DR detection models. For this study, a novel domain adaptation method (multi-model domain adaptation) is developed for unsupervised DR classification in unlabeled retinal images. At the same time, it only exploits discriminative information from multiple source models without access to any data. In detail, we integrate a weight mechanism into the multi-model-based domain adaptation by measuring the importance of each source domain in a novel way, and a weighted pseudo-labeling strategy is attached to the source feature extractors for training the target DR classification model. Extensive experiments are performed on four source datasets (DDR, IDRiD, Messidor, and Messidor-2) to a target domain APTOS 2019, showing that MMDA produces competitive performance for present state-of-the-art methods for DR classification. As a novel DR detection approach, this article presents a new domain adaptation solution for medical image analysis when the source data is unavailable.

## 1 Introduction

Diabetic retinopathy (DR) is a complication of diabetic patients and a significant cause of blindness globally among the working population (Antonetti et al., 2021). There are 451 million suffering from DR in the world, and this is projected to increase to 639 million in 2045 (Cho et al., 2018). In diabetics, blood is provided to all retina layers through micro blood vessels that are sensitive to unrestricted blood sugar levels. DR may cause no symptoms or only mild vision problems at first, but it can cause blindness eventually. When substantial glucose or fructose is collected in the blood, blood vessels begin to collapse due to insufficient oxygen supply to the cells. Occlusion in these blood vessels can cause serious eye damage. As a result, metabolic rate decreases, and abnormal blood vessels accumulate in DR (Dai et al., 2021). Microaneurysms (MAs) are the early signs of DR, which cause changes in the size (swelling) of the blood vessels. Moreover, hemorrhages (HMs), exudates (EXs), and abnormal blood vessel growth are the symptoms of DR. The International Clinical Diabetic Retinopathy (ICDR) scale is one of the most commonly used clinical scales and is composed of five levels of DR: normal, mild, moderate, severe and proliferative (Bodapati et al., 2021). Generally, diabetic retinopathy is divided into referable diabetic retinopathy (RDR) and non-referable diabetic retinopathy (NRDR).

Blindness can be completely avoided by early diagnosis. Annual regular clinical examination for diabetics is strongly recommended, especially for middle-aged and older adults (Mohamed et al., 2007; Ferris, 1993). Nevertheless, researchers find that a considerable number of people with diabetes failed to have annual eye examinations due to very mild symptoms, long examination time, and a shortage of ophthalmologists (Owsley et al., 2006; MacLennan et al., 2014; Chou et al., 2014). Therefore, it is necessary to adopt automatic DR diagnosis methods to lighten the workload on eye specialists and shorten the detection time, making patients understand the condition and get treatment in time.

Artificial intelligence (AI) is a popular technique for computer-aided automatic DR diagnosis to overcome these obstacles and deep learning has achieved progress in biomedical image analysis (Meng et al., 2021b; Preston et al., 2021; Meng et al., 2021a). Yoo and Park (2013) utilized ridge, elastic net, and LASSO to perform validation on 1052 DR patients. Roychowdhury et al. (2013) proposed a novel two-step approach for DR detection, where non-lesions or normal images are rejected in the first step, and bright and red lesions are classified as hard exudates and hemorrhages, respectively in the second step. In addition to the machine learning methods, the deep learning method becomes very popular in DR screening in recent years. For instance, Vo and Verma (2016) used a deep neural network improved upon GoogLeNet and VGGNet for DR recognition, aiming to learn fine-grained features of retinal images. Moreover, He et al. (2020) combined two attention blocks with a backbone network to solve the imbalanced DR data distribution problem and capture more detailed lesion information, respectively. Ai et al. (2021) proposed an algorithm adopting deep ensemble learning and attention mechanism to detect DR. However, both traditional machine learning methods (Yoo and Park, 2013; Roychowdhury et al., 2013) and supervised deep learning methods (Vo and Verma, 2016; He et al., 2020; Mohamed et al., 2021; Ai et al., 2021) require a large amount of labeled retinal images to train their models, which fail to new data from other domains. As an effective solution, domain adaptation always requires source data, which is usually difficult to access in practical applications because of the strict privacy rules in medical image management agencies.

**FIGURE 1:**
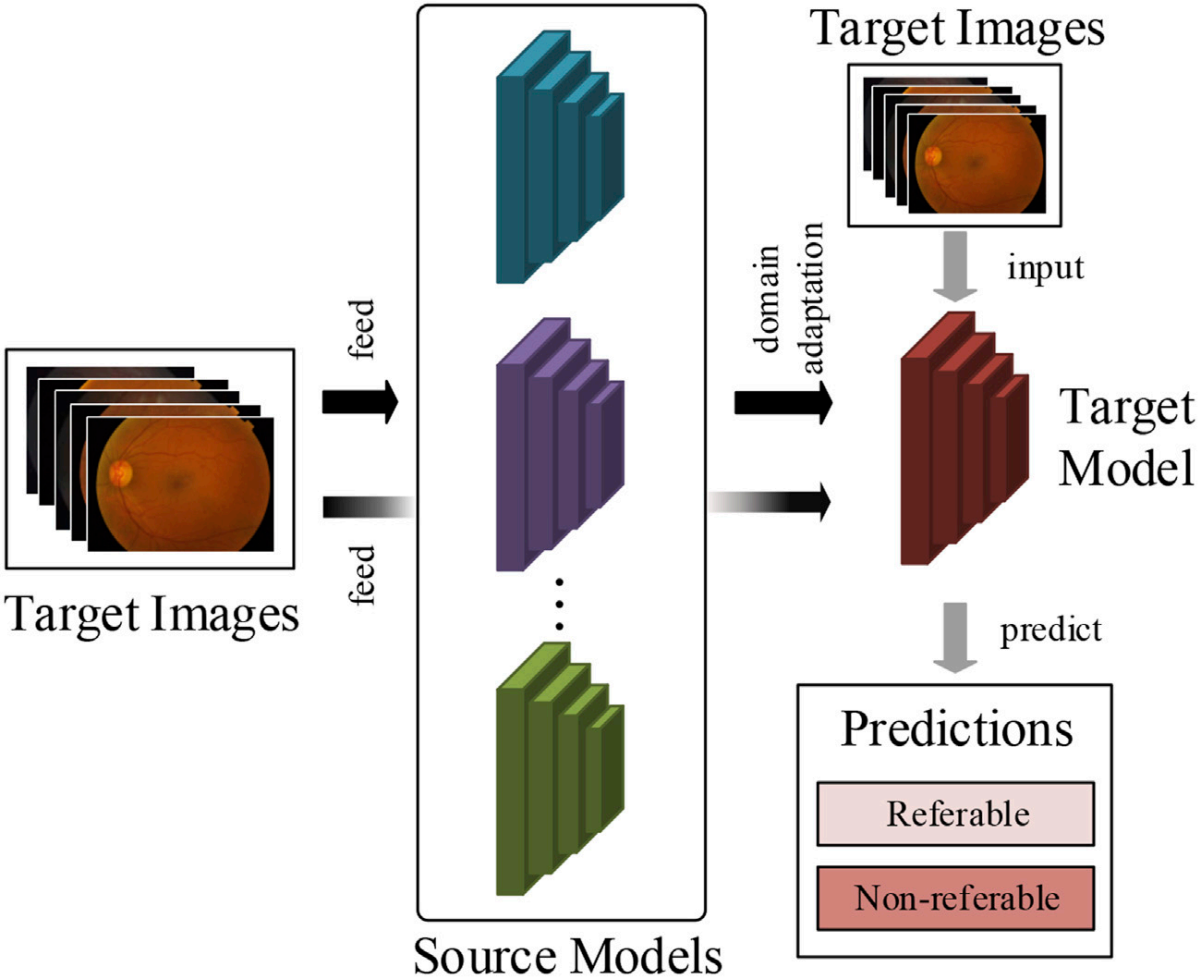
The work flow of our method. We train the target prediction model to simply use pre-trained multiple source models and unlabeled target retinal images.

To tackle this critical problem in supervised deep learning methods, this article attempts to develop a multi-model domain adaptation (MMDA) to conduct transfer learning for DR classification without access to source data. As shown in Figure 1, the proposed method can sufficiently utilize the knowledge of source models and unlabeled target images to improve the DR detection performance.

In the MMDA framework, the target model is initially parameterized, and the trained source models are provided. We propose a model weight determination module to estimate the importance of each source model by measuring the average distance between two retinal feature groups extracted from the source models and target model. This module is optimized by a weight determination loss to output realistic model weights in target feature learning. By using the weights of source models, the pseudo label of the target images is obtained in a feature-level clustering-based way. Finally, we optimize the target model by cross-entropy loss and information maximization loss to guarantee the performance of diabetic retinopathy detection.

To evaluate the performance of MMDA, we conduct extensive experiments on five publicly available retinal image datasets: DDR, IDRiD, Messidor, Messidor-2, and APTOS 2019, obtaining excellent performance without access to source data. The results demonstrate that our proposed method can effectively complete the DR diagnosis task with only unlabeled target data.

## 2 Materials and Methods

### 2.1 Data Acquisition

In order to validate our method for diabetic retinopathy diagnosis, we trained four source models from publicly available datasets (DDR, IDRiD, Messidor, and Messidor-2) and employed APTOS 2019 as the target domain.

DDR dataset (Diabetic retinopathy detection, 2015) involves 12,522 fundus images from a 45° field of view. In detail, it has 6,266 normal fundus images and 6,256 abnormal samples. Moreover, the class distribution of the dataset is imbalanced in that the normal images are more than the abnormal data.

The IDRiD dataset (Porwal et al, 2018) contains 516 fundus images which were captured by an ophthalmologist from an Indian eye clinic. It provides adequate quality and clinically relevant fundus images with ground truths.

The Messidor dataset (Decencière et al., 2014) is a publicly available diabetic retinopathy dataset provided by the Messidor program partners, which consists of 1,200 retinal images, and for each image, two grades, retinopathy grade, and risk of macular edema, are provided.

The Messidor-2 dataset (Decencière et al., 2014) has been globally used by researchers for DR detection algorithm analysis, which is an extension of Messidor. It contains 1,748 retinal images of 874 examinations. Although there are no official annotations for this dataset, the third-party grades for 1,744 out of the 1,748 images adjudicated by a panel of three retina specialists are available for researchers (Messidor-2 dr grades, 2018).

The APTOS 2019 dataset (Khalifa et al., 2019) is the most recent publicly available Kaggle dataset from the APTOS Blindness Detection competition on Kaggle for DR detection. It contains 3,662 labeled fundus photography images.

The above datasets are graded into five stages from 0 to 4 for no DR, mild DR, moderate DR, severe DR, and proliferative DR, respectively, according to the ICDR severity scale. The label distribution of the datasets and the division of the referable and non-referable DR are shown in Table 1. Moreover, the APTOS 2019 dataset is regarded as the target domain, and the other four datasets are used as source datasets to train source models.

**TABLE 1:** Label distributions of DDR, IDRiD, Messidor, Messidor-2, and APTOS 2019 datasets.

Dataset	Type	Non-referable			Referable	
		No	Mild	Moderate	Severe	Proliferative
DDR	Source	6,266	630	4,477	236	913
IDRiD	Source	168	25	168	93	62
Messidor	Source	546	153	247	254	—
Messidor-2	Source	1,017	270	347	75	35
APTOS 2019	Target	1,805	370	999	193	295

### 2.2 Data Preprocessing

When collecting retinal images, the differences in lighting conditions and camera types may cause a large data inconsistency (Graham, 2015). Data preprocessing mitigates noise and enhances image details, reducing inconsistency and playing a significant role in improving performance.

**FIGURE 2:**
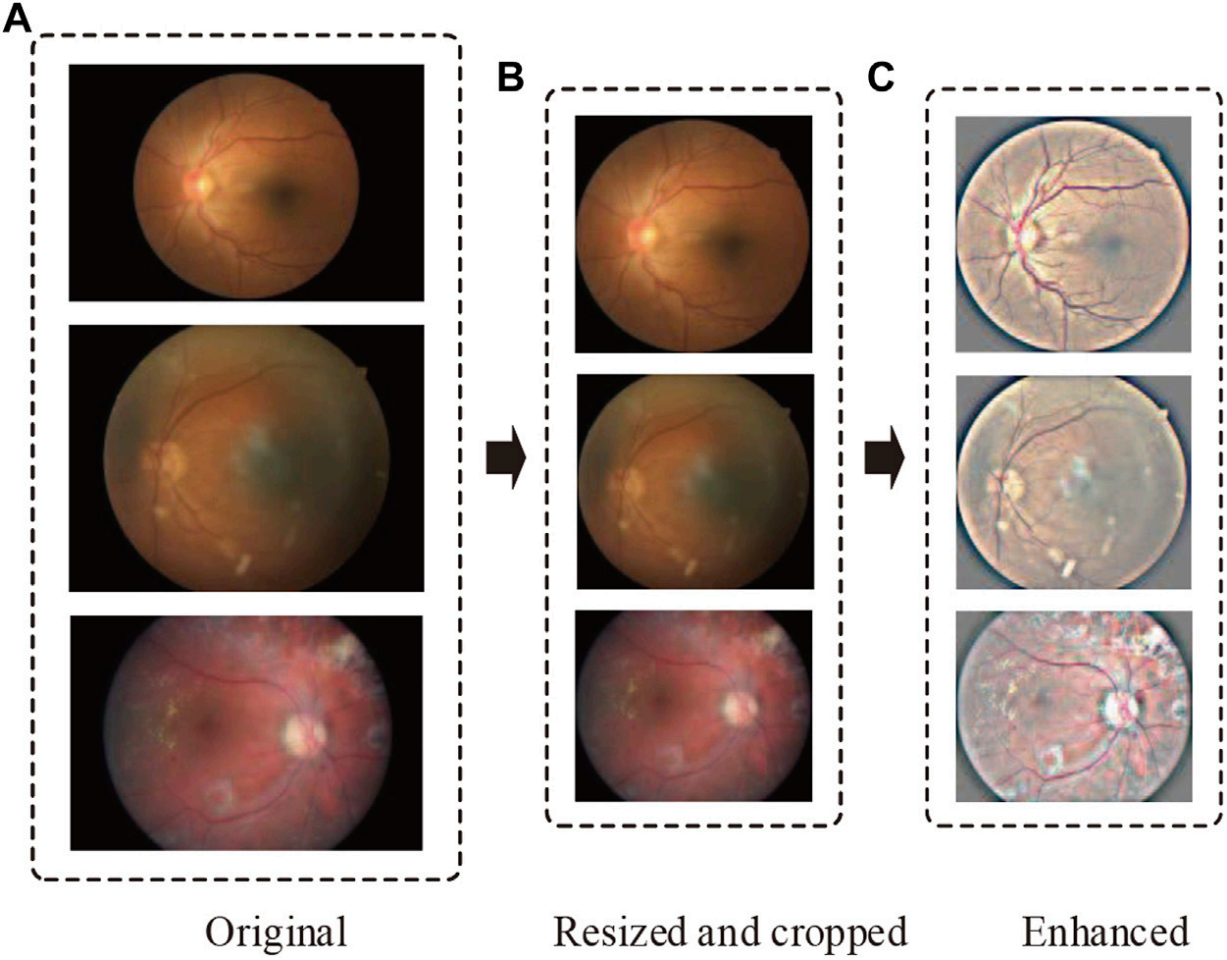
Representative retinal images adopting our preprocessing techniques. From top to bottom, the representative images are sampled from no DR, moderate DR, and proliferative DR, respectively. The parts **(A–C)** denote the original, resized and cropped, and enhanced retinal images.

In order to eliminate these negative effects and make data consistent, we perform data preprocessing in the following two steps (Figure 2):

Step 1: Resize and crop.

Considering various resolutions of retinal images in different datasets, we resize all images to 1,024 pixels if their width or height is bigger than that size. Then, we crop as much of the black space by identifying the center and radius of the circle in the retinal images.

Step 2: Image enhancement.

In DR detection, the observation of hard exudates, hemorrhages, and cotton wools is significant for eye specialists to diagnose. However, the variations of brightness and resolution not only make ophthalmologists produce misdiagnoses but also make it difficult for a model to compose robust features. To address this problem, we perform image enhancement after resizing and cropping by the following formula:

$$I_{o}\left(x,y;\sigma \right)=\lambda G\left(x,y;\sigma \right)*I\left(x,y\right)+\omega I\left(x,y\right)+\delta$$ (1)

where *I* (*x*, *y*) denotes the input retinal image, *G* (*x*, *y*; *σ*) is a Gaussian filter with standard deviation *σ*, “∗” represents the convolution operator. *λ*, *ω* and *δ* are manually adjusted variables. In our study, *λ*, *ω* and *δ* are set to 4, -4 and 128, respectively. By improving image contrast with Eq. 1, the lesion area is easier to distinguish.

### 2.3 Multi-Model Domain Adaptation Architecture

This subsection elaborates on our proposed MMDA method, which aims to address the central problem that the labeled image data cannot always be obtained in automatic DR detection.

#### 2.3.1 Overview

**FIGURE 3:**
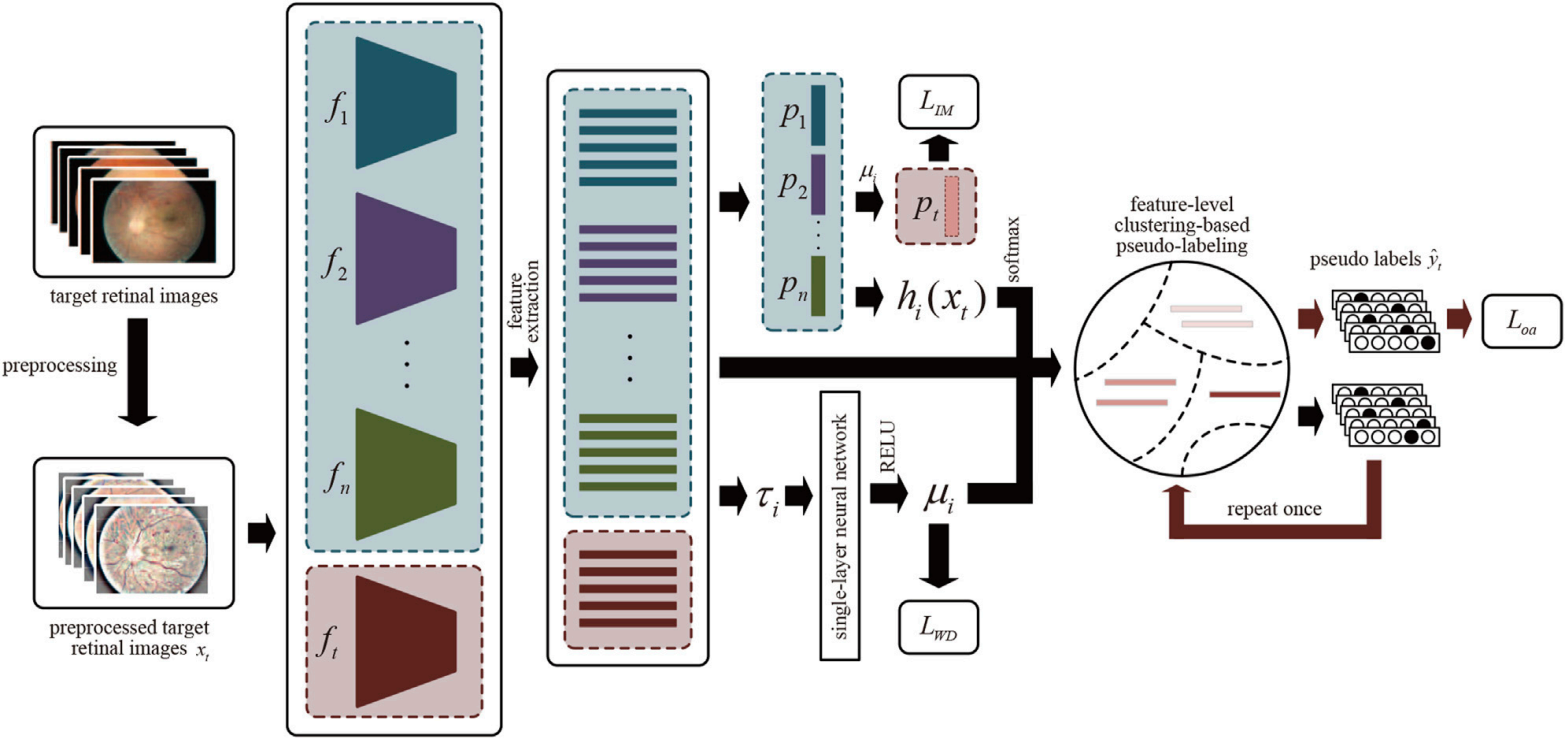
The overview of MMDA architecture. After preprocessing, we obtain the features of target retinal images by source models $f_{i}{|}_{i=1}^{n}$ and target model *f*_*t*_, and calculate the weight of each model *μ*_*i*_ using the single-layer neural network. The output of the target classifier is defined by the source classifiers with fixed parameters. Pseudo labels ${\hat{y}}_{t}$ for each retinal image *x*_*i*_ are obtained after the process of feature-level clustering-based pseudo-labeling.

Domain adaptation is one of the branches of transfer learning in computer science. For a vanilla multi-source unsupervised domain adaptation task, we have *n* source domains with fundus images, and $N_{s}^{i}$ labeled samples ${\left\{x_{i}^{j},y_{i}^{j}\right\}}_{j=1}^{N_{s}^{i}}$ from the *i*th source domain are given, where $x_{i}^{j}\in X_{i}$ , $y_{i}^{j}\in Y_{i}$ , and also *N*_*t*_ unlabeled retinal images ${\left\{x_{t}^{j}\right\}}_{j=1}^{N_{t}}$ from the target domain $D_{t}$ where $x_{t}^{j}\in X_{t}$ . Domain adaptation aims to obtain a target model to predict the labels ${\left\{y_{t}^{j}\right\}}_{j=1}^{N_{t}}$ , where $y_{t}^{j}\in Y_{t}$ . Here, the goal of MMDA is to learn a target prediction model for function $h_{t}:X_{t}\to Y_{t}$ and infer ${\left\{y_{t}^{i}\right\}}_{i=1}^{N_{t}}$ , with only ${\left\{x_{t}^{i}\right\}}_{i=1}^{N_{t}}$ and the source prediction models for function: $h_{i}:X_{i}\to Y_{i}$ available. Note that, only trained source models can be utilized, without access to their data. Figure 3 illustrates the overview of our MMDA model for referable DR detection.

Suppose that we have multiple trained source models for DR classification and an ImageNet pre-trained target model. Each model contains two modules: the feature encoding module $f_{i}:X_{i}\to R^{d}$ and the classifier module $p_{i}:R^{d}\to R^{K}$ , i.e., *h*_*i*_(*x*) = *p*_*i*_ (*f*_*i*_(*x*)). Here, *d* is the dimension of the feature, and *K* represents the number of categories. We extract the features of retinal images using the source and target feature encoding modules firstly. A single-layer neural network is integrated to determine the weights of each source model using euclidean distance and weight determination loss. By employing the weights and source classifiers, target prediction logits are obtained. Then the pseudo labels ${\hat{y}}_{t}$ of the target images are generated in a feature-level clustering-based way. Finally, the whole network is optimized using cross-entropy loss and information maximization loss to make the target feature encoding module has excellent DR diagnosis capability.

#### 2.3.2 Source Model Generation

We consider producing several source backbone pre-trained source models, i.e., *h*_*i*_ = *p*_*i*_◦*f*_*i*_ (*i* = 1, 2, ⋯*n*), by optimizing them using the following cross-entropy loss:

$$\begin{array}{cc} L_{\mathrm{src}}^{i} & \left(f_{i};X_{i},Y_{i}\right)=-E_{\left(x_{i},y_{i}\right)\in X_{i}\times Y_{i}}\overset{K}{\underset{k=1}{\sum }}q_{k}\log\sigma_{k}\left(h_{i}\left(x_{i}\right)\right) \end{array}$$ (2)

where $\sigma_{k}\left(a\right)=\frac{\exp\left(a_{k}\right)}{\sum_{i}\exp\left(a_{i}\right)}$ denotes the *k*th element in the softmax output of a *K*-dimensional vector *a*, and *q* is the one-hot encoding of *y*_*i*_ where *q*_*k*_ is set to “1” if *y*_*i*_ is the *k*th class and the rest is set to “0”. In order to learn more discriminative feature representations and further enhance the following target data alignment, we adopt the label smoothing technique because it prevents the model from becoming over-confident thus improving generalization and performance (Müller et al., 2019). With label smoothing, the objective loss function is modified as below:

$$\begin{array}{cc} L_{\mathrm{src,ls}}^{i} & \left(f_{i};X_{i},Y_{i}\right)=-E_{\left(x_{i},y_{i}\right)\in X_{i}\times Y_{i}}\overset{K}{\underset{k=1}{\sum }}q_{k,ls}\log\sigma_{k}\left(h_{i}\left(x_{i}\right)\right) \end{array}$$ (3)

where *q*_*k*,*ls*_ = (1 − *α*)*q*_*k*_ + *α*/*K* represents the smoothed label and *α* is the smoothing factor which is set to 0.1 experientially.

#### 2.3.3 Information Maximization Loss for Target Model

Due to the source classifier modules encoding the distribution information of unseen source data, our framework is proposed to learn the domain-specific feature encoding module while the source classifier modules are fixed. Specifically, MMDA employs the weighted source classifier modules during the target model learning process:

$$p_{t}\left(\cdot \right)=\overset{n}{\underset{i=1}{\sum }}\mu_{i}p_{i}\left(\cdot \right)$$ (4)

where *μ*_*i*_ is the weight for the *i*th source model, which will be explained in detail in the following subsection.

In essence, our goal is to obtain an optimal target feature extractor $f_{t}:X_{t}\to R^{d}$ for target retinal images, in order that the extracted target features can match source distributions well. However, it is noteworthy that the source images are not accessible in our study. As a result, there’s no way to perform feature-level alignment since it is unfeasible to estimate the source distribution in the absence of source data. We look at the problem from a different angle that the expected output logits of the target model should seem like one-hot encoding but differ from each other if the domain gaps are mitigated. To this end, we employ the information maximization (IM) loss (Hu et al., 2017), which enhance the certainty and diversity of target outputs. Specifically, we optimize *f*_*t*_ by IM loss *L*_*IM*_ that consists of two objective functions *L*_*ce*_ and *L*_div_:

$$\begin{array}{cc} L_{ce}\left(f_{t};X_{t}\right) & =-E_{x_{t}\in X_{t}}\overset{K}{\underset{k=1}{\sum }}\sigma_{k}\left(h_{t}\left(x_{t}\right)\right)\log\sigma_{k}\left(h_{t}\left(x_{t}\right)\right)\\ L_{\mathrm{div}}\left(f_{t};X_{t}\right) & =\overset{K}{\underset{k=1}{\sum }}P_{k}\log P_{k}=D_{KL}\left(P,\frac{1}{K}1_{K}\right)-\log K\\ L_{IM} & =Lce+\beta L_{\mathrm{div}} \end{array}$$ 5)

where *h*_*t*_(*x*) = *p*_*t*_ ( *f*_*t*_(*x*)) is the *K*-dimensional output logits of each retinal images, **1**_*K*_ is an all-ones vector with *K* elements, and $P=E_{x_{t}\in X_{t}}\left[\sigma \left(f_{t}\left(x_{t}\right)\right)\right]$ represents the average output probabilities of the whole target domain, *β* is the balance factor. Information maximization would work better than conditional entropy minimization (Grandvalet and Bengio, 2005) commonly used in traditional domain adaptation works, since it can circumvent the trivial solution where all unlabeled fundus images have the same one-hot encoding via the fair diversity-promoting objective function *L*_div_.

#### 2.3.4 Model Weight Determination

In the MMDA framework, a robust target feature encoder is learned by bridging the domain gap between each source domain and the target domain. However, the feature discrepancies between each source domain and target domain are different. To measure the feature discrepancies, we propose a Model Weight Mechanism (MWM). Precisely, we first calculate the average Euclidean Distance between the *i*th source domain and the target domain:

$$\tau_{i}=\frac{1}{N_{t}}\overset{N_{t}}{\underset{j=1}{\sum }}|f_{i}\left(x_{j}\right)-f_{t}\left(x_{j}\right)|$$ (6)

The closer the *τ*_*i*_ is, the more important the source model, i.e., the greater the weight is. To this end, we integrate a single-layer neural network, which is parameterized by a weight vector $w=\left(w_{1},w_{2},\cdots w_{d}\right)\in R^{d\times 1}$ . Formally, we learn model weight *μ*_*i*_ as

$$\mu_{i}=\frac{\exp\left(ReLU\left(w^{T}\tau_{i}\right)\right)}{\sum_{j=1}^{N}\exp\left(ReLU\right.\left(w^{T}\tau_{j}\right)}$$ (7)

where ReLU(⋅) = max (0, ⋅) is an activation function, which guarantees the nonnegativity of *μ*_*i*_. The role of the above softmax operation is to guarantee the model weight satisfy the following property:

$$\overset{n}{\underset{i=1}{\sum }}\mu_{i}=1,\mu_{i}\geq 0$$ (8)

We optimize the weight vector of the single-layer neural network *w* by minimizing the following loss function:

$$L_{WD}\left(w;X_{t}\right)=\frac{1}{N_{t}}\overset{n}{\underset{i=1}{\sum }}\overset{N_{t}}{\underset{j=1}{\sum }}\mu_{i}{\left\|f_{i}\left(x_{j}\right)-f_{t}\left(x_{j}\right)\right\|}_{2}^{2}$$ (9)

That is, a larger distance ${\left\|f_{i}\left(x_{j}\right)-f_{t}\left(x_{j}\right)\right\|}_{2}$ between features *f*_*i*_(*x*) and *f*_*t*_(*x*) enforces a smaller value *μ*_*i*_.

#### 2.3.5 Feature-Level Clustering-Based Pseudo-Labeling

The role of IM loss in Eq. 5 is to enforce the similarity of one-hot encoding output. Therefore, an accurate prediction network is crucial to reduce this impact. For this purpose, a pseudo-labeling strategy at the feature level is applied for better supervision during the adaptation process.

First, the weighted features centroid of target retinal images for each class is obtained, similar to weighted k-means clustering.

$$m_{k}^{\left(0\right)}=\frac{\sum_{i=1}^{n}\sum_{x_{t}\in X_{t}}\mu_{i}\sigma_{k}\left(h_{i}\left(x_{t}\right)\right)f_{i}\left(x_{t}\right)}{\sum_{x_{t}\in X_{t}}\sigma_{k}\left(h_{i}\left(x_{t}\right)\right)}$$ (10)

These centroids can robustly and more reliably characterize the distribution of different categories within the target domain. Then, the pseudo labels can be attained via the nearest centroid classifier:

$${\hat{y}}_{t}=\underset{k}{\mathrm{arg min}}D_{c}\left(f_{t}\left(x_{t}\right),m_{k}^{\left(0\right)}\right)$$ (11)

where $D_{c}\left(a,b\right)=1-\frac{a\cdot b}{\left\|a\|\|b\right\|}$ represents the cosine distance between vector *a* and *b*.

Based on generated ${\hat{y}}_{t}$ previously , new centroids $m_{k}^{\left(1\right)}$ and pseudo labels are computed:

$$\begin{array}{cc} m_{k}^{\left(1\right)} & =\frac{\sum_{i=1}^{n}\sum_{x_{t}\in X_{t}}1_{\left[{\hat{y}}_{t}=k\right]}\mu_{i}f_{i}\left(x_{t}\right)}{\sum_{x_{t}\in X_{t}}1_{\left[{\hat{y}}_{t}=k\right]}}\\ {\hat{y}}_{t} & =\underset{k}{\mathrm{arg min}}D_{c}\left(f_{t}\left(x_{t}\right),m_{k}^{\left(1\right)}\right) \end{array}$$ (12)

We refer to ${\hat{y}}_{t}$ in Eq. 12 as the final pseudo labels.

To sum up, given *n* source models *h*_*i*_ = *p*_*i*_◦*f*_*i*_ (*i* = 1, 2, ⋯*n*) and the final pseudo labels ${\hat{y}}_{t}$ generated from Eq. 12, MMDA fixes the parameters of sources classifiers, $p_{t}\left(\cdot \right)=\sum_{i=1}^{n}\mu_{i}p_{i}\left(\cdot \right)$ and optimizes the feature extractor *f*_*t*_ with the overall loss as:

$$\begin{array}{c} L_{oa}\left(f_{t}\right)=L_{IM}-\gamma E_{\left(x_{t},{\hat{y}}_{t}\right)\in X_{t}\times {\hat{Y}}_{t}}\overset{K}{\underset{k=1}{\sum }}1_{\left[k={\hat{y}}_{t}\right]}\log\sigma_{k}\left(p_{t}\left(f_{t}\left(x_{t}\right)\right)\right) \end{array}$$ (13)

where *γ* > 0 is a balancing hyper-parameter. The whole implementation of MMDA model is shown in Algorithm 1.

Algorithm 1 Pseudo-code of MMDA training process
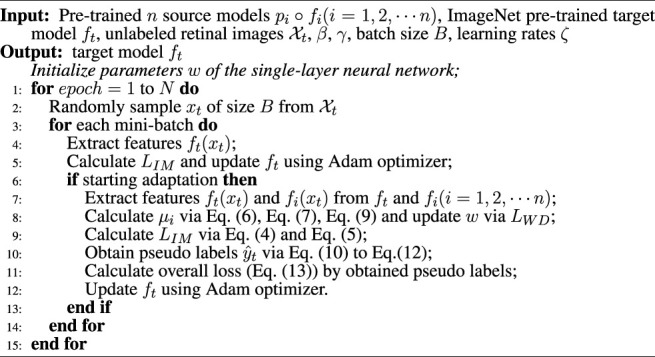

### 2.4 Implementation Details

In the experiments we first train the source models by corresponding source retinal datasets, and each model is designed following ResNet-50 (He et al., 2016). As for the target model, it also employs ResNet-50, initialized by pre-trained parameters from ImageNet (Deng et al., 2009). We perform data argumentation by applying random horizontal flips, vertical flips, and random rotation to prevent overfitting. The input size of the MMDA is 224 × 224. We trained 40 epochs for all the source models using the Stochastic Gradient Descent (SGD) optimization algorithm (Kingma and Ba, 2014) with a learning rate decay factor of 1*e* ^−4^. The learning rates for DDR, IDRiD, Messidor, and Messidor-2 datasets are 5 × 10^–3^ equally. For the target training, we adopt a mini-batch SGD with momentum 0.9, weight decay 1*e* ^−4^, and learning rate *ζ* = 1*e* ^−2^. The balance factor for IM loss *β* and the overall loss *γ* are set to 0.3 and 0.3, respectively. In addition, a batch size of 64 is set for the entire experimental process. MMDA is implemented on two NVIDIA RTX 2080Ti GPUs with 2 × 11 GB RAM using the PyTorch framework.

To validate the effectiveness of MWM (Section 2.3.4), we adjust *μ*_*i*_ in Eqs 4, 10, 12. Moreover, hyper-parameters *β* in *L*_*IM*_ and *γ* are fine-tuned to analyze their influence on DR detection performance. Details are described in Section 3.3 and Section 3.4. Note that we do not integrate model weight mechanism and pseudo-labeling into the target model until training the target model several epochs with IM loss. That means we attach the model weight and pseudo-labeling modules when the target model has a certain diagnosing capability.

## 3 Results

### 3.1 Evaluation Metrics

To measure the performance of the MMDA model, we employ accuracy and sensitivity as the measurements. The accuracy can be defined as the percentage of correctly classified images. Sensitivity measures the ability of a test to correctly identify samples with referable DR, which is an effective metric to measure the DR diagnosis.

This metric is calculated as follows. First, we compute the accuracy by $\frac{\text{TP}+\text{TN}}{\text{TP}+\text{FP}+\text{TN}+\text{FN}}$ , where TP is the correctly predicted positive samples, TN denotes the correctly predicted negative images, and FP represents the false predicted positive samples and FN means the false predicted negative images. For the sensitivity, it follows the formula,

$$\text{sensitivity}=\frac{\text{TP}}{\text{TP}+\text{FN}}$$ (14)

### 3.2 Performance Compared With Supervised Learning Methods

We first compare MMDA with the existing supervised learning methods on the APTOS2019 dataset. Specifically, Xie et al. (2017) present a simple, highly modularized network architecture for image classification, which is often employed in DR detection; Vives-Boix and Ruiz-Fernández (2021) conducted automated detection of DR by directly interfering in both learning and memory by reinforcing less common occurrences during the learning process; Narayanan et al. (2020) proposed a hybrid machine learning architecture to detect and grade the level of diabetic retinopathy; Farag et al. (2022) proposed an automatic deep-learning-based model for severity detection by utilizing a single color fundus photograph. From Table 2, it is observed that MMDA achieves approving results with 90.6% accuracy and 98.5% sensitivity. Our method performs a relatively excellent accuracy compared with the compared methods, which only remains a distance of 2.2%, and it presents the second-best sensitivity of 98.5%, only weaker than Narayanan et al. (2020). Although the results of MMDA are lower than these supervised learning methods, huge amounts of labeled data are essentially required in their training process. In contrast, we train MMDA simply to utilize unlabeled retinal images and obtain satisfactory performance, showing the superiority of our framework for DR diagnosis.

**TABLE 2:** Accuracy and sensitivity of MMDA for diabetic retinopathy diagnosis compared with state-of-the-art supervised learning approaches on the APTOS 2019 dataset.

Method	Accuracy (%)	Sensitivity (%)
Xie et al. (2017)	92.8	86.8
Vives-Boix and Ruiz-Fernández (2021)	94.5	90.0
Narayanan et al. (2020)	98.4	98.9
Farag et al. (2022)	97.0	97.0
MMDA (Ours)	90.6	98.5

### 3.3 Performance Analysis on Model Weight Mechanism

We design a novel model weight mechanism (MWM) to assign a learnable weight to each model. To verify the effect of the MWM, we perform ablation studies to analyze the MWM for the source classifier modules and the pseudo-labeling process using different backbones.

MWM for source classifier modules: We fix the source classifier modules, so we can fully utilize the source distribution information in the modules when the source data is not available. Meanwhile, the discrepancy between each source domain and the target domain cannot be ignored. Specifically, we integrate the weight mechanism into the classifiers by Eq. 4. To verify the effect of MWM in utilizing the source distribution information, we conduct a study using Average-weighted Classifier Multi-model Domain Adaptation (ACDA), which is the MMDA model with $\mu_{i}=\frac{1}{n}\left(i=1,2,\cdots n\right)$ in Eq. 4. As shown in Table 3, we obtain accuracy and sensitivity of 0.873 and 0.965 for VGG-16, 0.880 and 0.972 for RestNet-50, with accuracy drops of 2.8%, 2.6% for VGG-16 and ResNet-50, respectively, which demonstrates that MWM contributes huge effectiveness on multi-model source distribution learning.

**TABLE 3:** The DR classification results of MMDA with different backbones on the APTOS 2019 dataset.

Backbone	Method	Accuracy	Sensitivity	*δ* Accuracy (%)
VGG-16	ACDA	0.873	0.965	*↓*2.8
	APDA	0.882	0.973	*↓*1.9
	MMDA	**0.901**	**0.980**	-
ResNet-50	ACDA	0.880	0.972	*↓* 2.6
	APDA	0.902	0.960	*↓* 0.4
	MMDA	**0.906**	**0.985**	-

The best results are in bold.

MWM for pseudo-labeling: To evaluate the contribution of MWM in features centroid determination, we carry out an experiment named APDA, which is a modified MMDA with $\mu_{i}=\frac{1}{n}\left(i=1,2,\cdots n\right)$ in Eqs 10–12. With this setting, this model performs at accuracies of 0.882 and 0.902 for VGG-16 and ResNet-50 respectively. All results are lower than the original MMDA model. This is because the average model weight cannot determine accurate centroids of features, which results in incorrect pseudo labels and failing to bridge the domain gap between each source domain and target domain. With MWM, the importance of each source model can be determined, which helps to obtain a more accurate pseudo label and improve the model performance.

### 3.4 Performance Analysis on Hyper-Parameters

To further validate the effectiveness of each component in MMDA, we explore the influence of hyper-parameters on the performance of our model.

**The choice of** ***β*** **in** ***L***_***IM***_ **:** *β* is a balancing factor that adjusts the contribution of fair diversity-promoting objective *L*_div_. The DR classification performance of MMDA with *β* from 0.1 to 0.5 is shown in Table 4. As reported, both accuracy and sensitivity is improved with the increase of *β*, and MMDA achieves the best performance when *β* is set to 0.3. However, when we further increase the value of *β*, the results start to decrease. We consider that the high *β* value weakens the effect of *L*_*ce*_, which leads the decision boundary to go through the high-density region.

**TABLE 4:** DR classification results using different *β* on the APTOS 2019 dataset.

*β*	0.1	0.2	0.3	0.4	0.5
Accuracy	0.899	0.903	**0.906**	0.902	0.851
Sensitivity	0.984	0.962	**0.985**	0.987	0.986

The best results are in bold.

The choice of *γ*: *γ* is a balancing factor of the information maximization loss *L*_*IM*_ and the cross-entropy loss in the overall loss *L*_*oa*_. In this section, we investigate the effectiveness of this hyper-parameter. The results shown in Table 5 demonstrate that MMDA achieves the highest effectiveness when *γ* is set to 0.3. The cross-entropy loss in the overall loss *L*_*oa*_ acts as a guide of the target model. If *γ* is too small, the effect of the pseudo labels is reduced. If *γ* is too large, the generalization of the target model will be limited. In order to learn more discriminative features in the target domain and enhance the DR diagnosis performance of the model, it is necessary to adjust the best value of *γ*.

**TABLE 5:** DR classification results using different *γ* on the APTOS 2019 dataset.

*γ*	0.1	0.2	0.3	0.4	0.5
Accuracy	0.877	0.896	**0.906**	0.905	0.902
Sensitivity	0.976	0.982	**0.985**	0.966	0.963

The best results are in bold.

### 3.5 Visual Analysis of Model Performance

Furthermore, in order to prove the superiority of the MMDA framework for practical applications, the ROC curve, and t-SNE plot are adopted to visualize our model.

**FIGURE 4:**
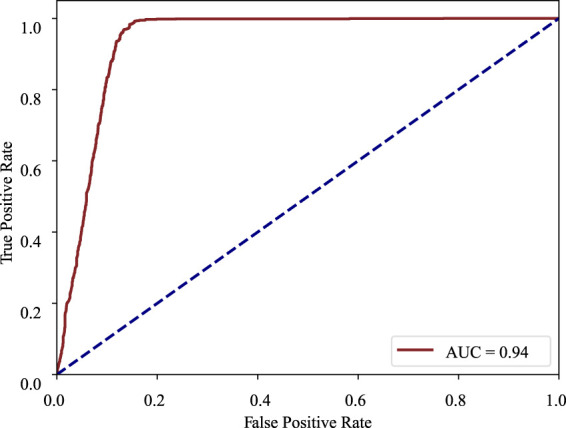
ROC curve of DR diagnosis on the APTOS 2019 dataset.

**FIGURE 5:**
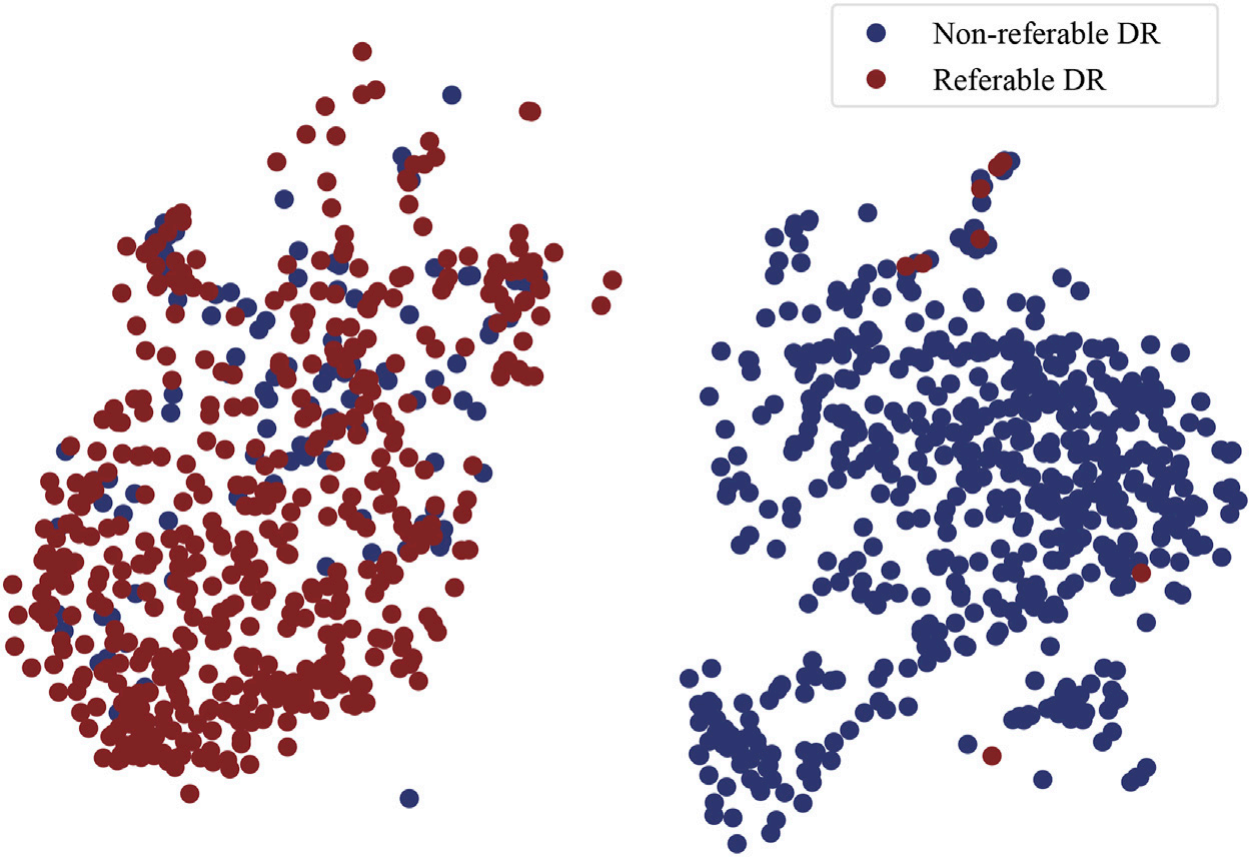
The t-SNE plot of DR classification on the APTOS 2019 dataset.

ROC curve: The receiver operating characteristic curve is a graphical plot that illustrates the diagnostic ability of a binary classifier system as its discrimination threshold is varied. The ROC curve is created by plotting the true positive rate (TPR) against the false positive rate (FPR) at various threshold settings. In the ROC curve, the closer the apex of the curve toward the upper left corner, the greater the discriminatory ability of the test. The ROC curve of MMDA for diabetic retinopathy classification is drawn in Figure 4, which obtains the area under the ROC curve of 0.94 and is above the diagonal and close to the point in the upper left corner, demonstrating that MMDA has a satisfying prediction performance.

t-SNE plot: t-Distributed Stochastic Neighbor Embedding (t-SNE) is a technique for dimensionality reduction that is particularly well suited for the visualization of high-dimensional data. It maps high-dimensional data to two or more dimensions suitable for human observation. In order to validate the effectiveness of MMDA, we perform a t-SNE plot using the target image features extracted by the trained target feature encoding module (*f*_*t*_). As shown in Figure 5, retinal images of non-referable DR and referable DR are well separated, because MMDA can learn discriminative features to detect referable diabetic retinopathy. The relatively clear boundaries in Figure 5 suggest that it is practical to train a robust prediction model using MMDA in the absence of labeled target data.

## 4 Discussion

Retinal images are usually used to build an automatic diabetic retinopathy diagnosis system (Gardner et al., 1996; Acharya et al., 2009; Ram et al., 2010; Gulshan et al., 2016; Lam et al., 2018; Jiang et al., 2019; Preston et al., 2021). However, whether using traditional machine learning methods (Gardner et al., 1996; Acharya et al., 2009; Ram et al., 2010) or deep supervised learning methods (Gulshan et al., 2016; Lam et al., 2018; Jiang et al., 2019; Preston et al., 2021), they all need a large amount of labeled data during training. In the biomedical image analysis field, labeling work is expensive and the privacy issue is highly sensitive. To tackle this challenge, we consider developing an unsupervised method that the DR diagnosis performance is excellent but labeled retinal images are unnecessary.

In this article, we present a novel MMDA that incorporates model weight mechanism into the MMDA technique. MMDA can be trained in an end-to-end manner with merely unlabeled target retinal images for DR classification. To the best of our knowledge, MMDA is the first attempt to automatically diagnose diabetic retinopathy by adopting an unsupervised domain adaptation technique with multiple source models. The main advantage of this article is that the MMDA can learn helpful knowledge only from source models without any source data, which can relieve the limitation of data privacy from different medical agencies.

Our proposed MMDA method aims to exploit the source knowledge and relationship between the source models and the target model, instead of learning from labeled retinal images directly, thus helping protect the patients’ privacy and no need to label images.

In order to fully explore the discrepancy between each source domain and target domain, we propose a model weight mechanism. By incorporating the mechanism into the source classifiers and feature-level clustering-based pseudo-labeling process, the diagnosis performance of the target model is improved.

Extensive experiments and ablation studies on the APTOS2019 dataset demonstrate that MMDA achieves competitive DR diagnosis performance in comparison with state-of-the-art supervised learning methods. However, the DR classification performance still has a distance from the advanced supervised methods due to the discrepancy between source and target models, especially for the invalid access to source data.

Model visualizations (Figures 4, 5) suggest that non-referable (grade 0, 1) and referable cases (from grade 2 to 4) can be diagnosed well. We will focus on the fine-grained classification of the DR grading task (Zheng et al., 2017) in the future.

## 5 Conclusion

When incorporating deep learning techniques in the automatic DR diagnosis system, time-consuming labeling work and privacy issues are critical problems. The present study is designed to exploit existing models and unlabeled retinal images for DR diagnosis to resolve these issues. Ablation studies show the effectiveness of our proposed modules, and the comparison with state-of-the-art supervised learning approaches demonstrates the superiority of our method. Moreover, model visualization indicates that our method can effectively diagnose non-referable and referable cases, with excellent diagnosing results.

## Data Availability

The original contributions presented in the study are included in the article material, further inquiries can be directed to the corresponding authors.
